# Sitagliptin-Dependent Differences in the Intensity of Oxidative Stress in Rat Livers Subjected to Ischemia and Reperfusion

**DOI:** 10.1155/2019/2738605

**Published:** 2019-10-31

**Authors:** Małgorzata Trocha, Małgorzata Krzystek-Korpacka, Anna Merwid-Ląd, Beata Nowak, Małgorzata Pieśniewska, Piotr Dzięgiel, Agnieszka Gomułkiewicz, Przemysław Kowalski, Dorota Diakowska, Adam Szeląg, Tomasz Sozański

**Affiliations:** ^1^Department of Pharmacology, Wroclaw Medical University, Jana Mikulicza-Radeckiego 2, 50-345 Wrocław, Poland; ^2^Department of Medical Biochemistry, Wroclaw Medical University, Chałubińskiego 10, 50-368 Wrocław, Poland; ^3^Department of Human Morphology and Embryology, Division of Histology and Embryology, Wroclaw Medical University, Chałubińskiego 6a, 50-368 Wrocław, Poland; ^4^Department of Physiotherapy, University School of Physical Education, I.J. Paderewskiego 35, 51-612 Wroclaw, Poland; ^5^Department of Pathomorphology and Oncological Cytology, Wroclaw Medical University, Borowska 213, 50-556 Wrocław, Poland; ^6^Division of Nervous System Diseases, Department of Clinical Nursing, Faculty of Health Sciences of Wroclaw Medical University, K. Bartla 5, 51-618 Wrocław, Poland

## Abstract

**Purpose:**

Ischemia/reperfusion (IR) is the main cause of liver damage after transplantation. We evaluated the effect of sitagliptin (STG) on oxidative stress parameters in the rat liver under IR.

**Methods:**

Rats were treated with STG (5 mg/kg) (S and SIR) or saline solution (C and CIR). Livers from CIR and SIR were subjected to ischemia (60 min) and reperfusion (24 h). During reperfusion, aminotransferases (ALT and AST) were determined in blood samples. Thiobarbituric acid reactive substances (TBARS), superoxide dismutase (SOD), catalase (CAT), paraoxonase-1 (PON1), glutathione peroxidase (GPx), and the mRNA expression of SOD1 were determined in liver homogenates after reperfusion. Different regions of livers were also histologically evaluated.

**Results:**

The PON1 activity was higher, and the TBARS level was lower in SIR than in CIR. There was an inverse relationship between TBARS and PON1 levels in the whole cohort. The GPx activity was lower in ischemic than in nonischemic groups regardless of the STG treatment. In SIR, the SOD1 activity was higher compared to that in CIR. In S, the expression of SOD1 mRNA was the highest of all examined groups and positively correlated with the SOD1 activity in the whole animal cohort. During IR aminotransferases, the activity in the drug-treated group was lower in all examined points of time. In drug-treated groups, the percentage of steatosis was higher than that in nontreated groups regardless of IR.

**Conclusions:**

The protective effect of STG on the rat liver, especially its antioxidant properties, was revealed under IR conditions.

## 1. Introduction

Ischemia/reperfusion (IR) is the main cause of liver injury that occurs during such procedure as transplantation or hepatectomy [1]. Initially, this damage is caused by ischemia, but further, it is aggravated by reperfusion. Among the many phenomena occurring in the IR, there is an excessive production of free radicals and the development of oxidative stress [2, 3]. Lipid peroxidation is one of the manifestations of oxidative imbalance accompanying rapid tissue reoxygenation and responsible for detrimental effects of IR. The process is initiated by the attack of reactive oxygen species (ROS) on double bonds of membrane phospholipids, glycolipids, and cholesterol. Malondialdehyde among others, are end products of lipid peroxidation. Lipid peroxidation, if not counteracted by antioxidants, leads to the deterioration of cellular membranes, loss of cell integrity, and cell death [4].

Superoxide dismutases (SODs) are antioxidant enzymes that convert superoxide radicals to oxygen and hydrogen peroxide [2]. There are three isoforms of SOD in mammals: SOD1, SOD2, and SOD3. SOD1, the major cytoplasmic isoenzyme which activity depends on the presence of the Cu and Zn, is expressed highly in selected tissues, mainly in the liver [5]. Hydrogen peroxide is reduced by selenium-containing enzyme—glutathione peroxidase (GPx) to water. Additionally, it is converted to water and molecular oxygen by catalase (CAT)—antioxidant enzyme located mainly in peroxisomes [6]. GPx is the most tightly associated with lipid peroxidation. In addition to preventing hydroxyl radical from forming, GPx is also responsible for two-electron reduction of hydroperoxides, primary products of lipid peroxidation, averting their one-electron reduction that facilitates propagation phase of the process [4]. Paraoxonase-1 (PON1) is a liver-synthesized esterase and lactonase characterized by broad substrate specificity. Best known is the enzyme form residing on HDL and involved in the protection of lipoproteins against lipid peroxidation. Apart from circulation, PON1 is present mainly in the liver where it participates in the inactivation of oxidative by-products, generated during biotransformation of xenobiotics in the microsomes [7]. Intracellular form of PON1 was also shown to involve in the stabilization of lipid membranes and the enhancement of their integrity under oxidative stress conditions [8]. Thiobarbituric acid reactive substances (TBARS) are the final lipid peroxidation product. The accumulation of malondialdehyde (MDA) occurs already in the ischemic phase and ROS-generating reperfusion exacerbating lipid peroxidation [9]. Due to inhibition of oxidative phosphorylation [10], ischemia accelerates processes, such as glycolysis and ketogenesis, which yield methylglyoxal as a by-product. Methylglyoxal is a glycoside factor initiating lipid peroxidation, leading to the formation of MDA [11]. Moreover, MDA can be synthesized enzymatically from thromboxane A2 [4], the synthesis of which is upregulated during ischemia [12].

Because extensive damage of the liver subjected to IR depends, among others, on the intensity of oxidative stress, the search for such substances that would enhance the antioxidant defense is justified. Sitagliptin (STG) belongs to a group of oral hypoglycemic drugs that, through inhibition of the dipeptidyl peptidase-4 (DPP-4) activity, prolong the half-life and thus action of incretins—glucose-dependent insulinotropic polypeptide (GIP) and glucagon-like peptide-1 (GLP-1) [13, 14]. STG is approved in more than 130 countries worldwide in monotherapy or in combination with other hypoglycemic drugs for the treatment of patients with type 2 diabetes. STG is generally well tolerated, with gently or moderately intensified adverse events [15]. The choice of STG could be based on the additional properties of this drug, such as antioxidative action, that have been reported in some works [16, 17].

The aim of this work was to evaluate the potential antioxidative and hepatoprotective properties of STG administered chronically to rats prior to liver IR procedure.

## 2. Materials and Methods

### 2.1. Animals

The study was carried out on Wistar male rats at the age of 2-3 months. Animals were housed in individual chambers in standard conditions (a 12 : 12 h light-dark cycle, humidity 45-60%, continuous ventilation, and the temperature maintained at 21–23°C).

#### 2.1.1. Ethical Approval and Informed Consent

All procedures performed in the study were in accordance with the ethical standards of the institutional and/or national research committee. All applicable international, national, and/or institutional guidelines for the care and use of animals were followed. The experiment protocol was approved by the 1^st^ Local Ethics Committee on the Animal Research of the Institute of Immunology and Experimental Therapy Polish Academy of Sciences in Wroclaw (# 80/2012 of December 5, 2012).

### 2.2. Chemicals

Sitagliptin (Januvia, tabl. 100 mg; MSD, Poland), heparin (Heparinum WZF, amp. 25000 U/5 ml; Polfa Warszawa, Poland), ketamine hydrochloride (Bioketan, Vetoquinol Biowet, Poland), medetomidine hydrochloride (Domitor, amp. 1 mg/ml, Orion Pharma, Finland), butorphanol tartrate (Morphasol, amp. 4 mg/ml, aniMedica GmbH, Germany), 0.9% sodium chloride solution (Polpharma S.A., Poland), and Ringer's solution (Polfa Lublin S.A., Poland) were used in the study.

### 2.3. Experimental Design

Following adaptation period, rats were divided randomly into 4 groups. In group C (*n* = 9) and CIR (*n* = 9), animals were not treated with STG. In group S (*n* = 8) and SIR (*n* = 10), animals received STG (5 mg/kg p.o.) once a day for 2 weeks prior to the surgical procedure. Livers from groups SIR and CIR were subjected to the IR procedure. To determine the initial activity of ALT and AST, blood samples were obtained from the tail vein after the STG treatment.

### 2.4. IR Procedure

After intramuscular injection of medetomidine hydrochloride (0.1 mg/kg), ketamine hydrochloride (7 mg/kg), and butorphanol tartrate (2 mg/kg), animals were subjected to midline laparotomy. In groups CIR and SIR, branches of the hepatic artery and portal vein were occluded with a microvascular clip, which caused 70% of the liver (median and left lateral lobes) to be ischemic. Rats were given heparin (200 U/kg) to prevent blood coagulation. The clip was removed after 60 min of ischemia to allow reperfusion for 24 h. At 2, 6, and 24 h of reperfusion, samples of blood were collected to determine the activity of aminotransferases. Once the experiment was terminated, livers were weighted and ischemic lobes were isolated. A part of the liver lobes was placed in the RNA*later* RNA Stabilization Reagent (Qiagen, Germany) and used for real-time PCR. Remaining ischemic liver tissue was homogenized, and supernatant was collected.

In S and C groups, rats underwent the same anesthesia and surgical procedure as in the ischemic groups (CIR and SIR), but after midline laparotomy, the branches of the portal vein and hepatic artery were not occluded. Blood samples were obtained at the same time points as in the case of ischemic groups. After 24 hours of reperfusion, the same lobes of livers were isolated as in ischemic groups. All surgical procedures with or without I/R were blindly performed by the same experienced team of researchers.

### 2.5. The Homogenization of Isolated Fragments of the Liver

Liver tissue fragments (400-500 mg) were homogenized 1 : 2 (*w*/*v*) in Tris-EDTA buffer pH 7.2 (10 mM Tris, 1 mM EDTA, 1 mM MgCl_2_, and 150 mM KCl) with 1% deoxycholate, 1 mM PMSF, and 1% Triton X-100, using ceramic lysing matrix beads and the FastPrep-24™ homogenizer (MP Biomedicals, LCC, CA, USA). Homogenates were centrifuged (14,000 × g, 10 min, 4°C), and supernatants were collected, aliquoted, and stored at -80°C. Prior to analyses, homogenate samples were diluted with Tris-EDTA buffer or respective assay buffers with optimal dilution factors established for each assay by serial dilutions.

### 2.6. Oxidative Stress Parameters and Biochemical Analyses

The concentrations of thiobarbituric acid reactive substances (TBARS) were determined with the thiobarbituric acid spectrophotometric assay [18] with the addition of butylated hydroxytoluene (Fluka, Switzerland). The activities of copper-zinc superoxide dismutase (SOD1) and GPx were measured using Superoxide Dismutase Assay Kits and Glutathione Peroxidase Assay Kits (Cayman Chemical, MI, USA), according to the manufacturer's instructions and expressed as units (U/g for SOD) or kilounits (kU/g for GPx) per gram of liver tissue. The CAT activity was measured according to the procedure described by Bartosz [19], in which the decrease in absorbance at 240 nm, caused by decomposition of hydrogen peroxide (H_2_O_2_), was assessed for one minute. One unit of an enzyme activity is defined as one millimole of degraded H_2_O_2_ per minute. The enzyme activity was expressed as kilounits per gram (kU/g) of liver tissue.

The PON1 activity was determined spectrophotometrically by measuring the rates of phenyl acetate (Sigma-Aldrich, St. Louis, MO) hydrolysis, according to the Arylesterase/Paraoxonase Assay Kit protocol (ZeptoMetrix Co., Buffalo, NY). The enzyme activity was expressed in kilounits per gram (kU/g) of liver tissue. One unit (U) of the enzyme activity was defined as one mmol of released phenol per 1 liter per minute in 25°C. The enzyme activity towards phenyl acetate (PON1 arylesterase activity) is considered as a surrogate for the enzyme concentration [20]. All measurements were conducted at least in duplicates, and technical replicates were averaged.

Serum activities of aminotransferases (ALT and AST) and protein concentration in homogenates were assayed using a commercial enzymatic method in a certified laboratory.

### 2.7. RNA Isolation and Reverse Transcription

Total RNA was isolated from the studied tissue samples with RNeasy Mini Kit (Qiagen, Hilden, Germany) according to the manufacturer's protocol. To eliminate genomic DNA contamination, on-column DNase digestion was performed using RNase-Free DNase Set (Qiagen, Hilden, Germany). Quantity and purity of RNA samples were assessed by measuring the absorbance at 260 and 280 nm with NanoDrop1000 Spectrophotometer (Thermo Fisher Scientific, Wilmington, DE, USA). First-strand cDNA was synthesized using the High Capacity cDNA Reverse Transcription Kit (Applied Biosystems, Carlsbad, CA, USA).

### 2.8. Real-Time PCR

The mRNA expression of SOD1 was determined by real-time PCR with 7900HT Fast Real-Time PCR System and TaqMan Gene Expression Master Mix (Applied Biosystems, Carlsbad, CA, USA). Beta-2 microglobulin (B2M) was used as reference gene. For the reactions, the following sets of primers and TaqMan probes were used: Rn00566938_m1 for SOD1 and Rn00560865_m1 for B2M (Applied Biosystems, Carlsbad, CA, USA). All reactions were performed in triplicates in standardized thermal cycling conditions, including polymerase activation at 50°C for 2 min, denaturation at 94°C for 10 min, and 40 cycles of denaturation at 94°C for 15 s followed by annealing and synthesis at 60°C for 1 min. The relative amount of SOD1 at the mRNA level (RQ) was calculated by the 2^-*∆∆*Ct^ method.

### 2.9. Histological Examination

Different regions of the isolated lobes of livers from ischemic and nonischemic groups were fixed in 10% formalin and embedded in paraffin. Sections of 4.5 *μ*m were made and stained with hematoxylin-eosin. Then, they were histologically evaluated under a light microscope for the severity of ischemic necrosis, degree of steatosis as percentage of the microscopic field (small cytoplasmic vacuoles containing lipids or single fat droplets displacing the nuclei of hepatocytes), neutrophil infiltration, and destruction of hepatic architecture.

## 3. Statistical Analysis

Data distribution and homogeneity of variances were tested using Kolmogorov-Smirnov and Levene tests, respectively. Normally distributed data (SOD1 activity and expression, CAT, and aminotransferases) were expressed as means ± SD and analyzed using one-way ANOVA with Bonferroni correction for multiple testing, followed by post hoc test. Nonnormally distributed data (TBARS, PON1, and GPx) were expressed as medians with interquartile range and analyzed using Kruskal-Wallis *H* test with the Conover post hoc analysis. Statistical analysis of the effect of the drug and time of reperfusion on the aminotransferase activity was performed using repeated measures ANOVA. Correlation analysis was conducted using Spearman (*ρ*) or Pearson (*r*) correlation tests, depending on the data distribution. All tests were two-tailed, and hypotheses were considered positively verified if *p* < 0.05. The analyses were performed using MedCalc Statistical Software version 17.4.4 (MedCalc Software bvba, Ostend, Belgium; https://www.medcalc.org; 2017) and Statistica (13.1).

## 4. Results

### 4.1. Aminotransferases

**Figure 1:**
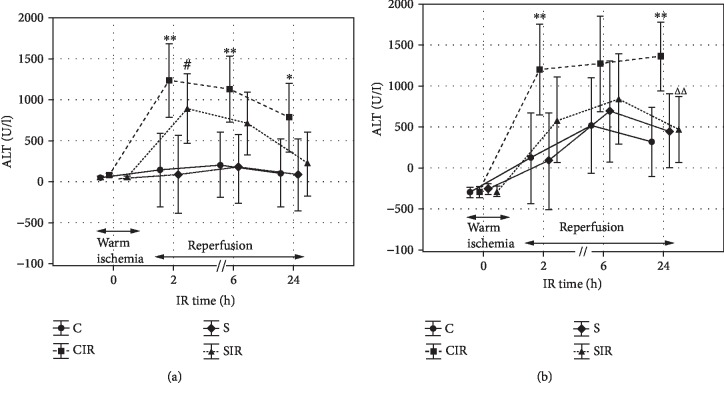
Influence of IR and STG treatment on activity of ALT (a) and AST (b). Values are presented as the mean + SD. Group C, nontreated and nonsubjected to IR; group CIR, nontreated and subjected to IR; group S, STG-treated and nonsubjected to IR; group SIR, STG-treated and subjected to IR. Specific comparisons: ^∗^*p* < 0.05 and ^∗∗^*p* < 0.01 (compared to C), ^#^*p* < 0.05 (compared to S), and ^ΔΔ^*p* < 0.01 (compared to CIR).

After 2 weeks of the STG administration, before surgical procedure, the activity of ALT in rats from treated groups (S+SIR) was significantly lower compared to nontreated groups (C+CIR) (*p* < 0.05). At 2 h of reperfusion, the significant increase in the ALT activity was noticed regardless of STG administration (CIR vs. C, *p* < 0.01 and SIR vs. S, *p* < 0.05). Values of ALT in the STG-treated ischemic group were insignificantly lower than in the nontreated group. Differences between AST activities were also significant between nonischemic and ischemic nontreated groups (CIR vs. C, *p* < 0.01). After 6 h of reperfusion, the activity of ALT was statistically higher in the nontreated ischemic group compared to the nonischemic group (CIR vs. C, *p* < 0.01). In STG-treated groups, this difference was close to the significance threshold (SIR vs. S, *p* = 0.06). After 24 h of reperfusion, the activity of aminotransferases was the highest in the ischemic nontreated group (CIR vs. C, *p* < 0.01 for AST and *p* < 0.05 for ALT). The increase in the aminotransferase activity in STG-treated groups was not significant. Hence, therein, in the case of ALT, that difference was close to the significance threshold (SIR vs. CIR, *p* = 0.05), and in the case of AST, that difference was significant (SIR vs. CIR, *p* < 0.01) (Figures 1(a) and 1(b)).

### 4.2. Parameters of Oxidative Stress

**Figure 2:**
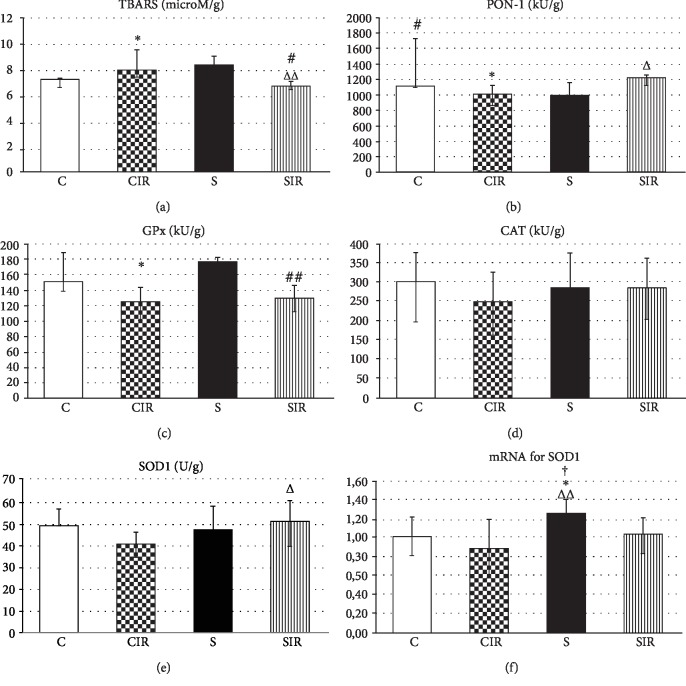
Influence of IR and STG treatment on TBARS (a), PON-1 (b), GPx (c), CAT (d), SOD1 (e), and mRNA for SOD1 (f) levels. Values are presented as the mean + SD. Group C, nontreated and nonsubjected to IR; group CIR, nontreated and subjected to IR; group S, STG-treated and nonsubjected to IR; group SIR, STG-treated and subjected to IR. Specific comparisons: ^∗^*p* < 0.05 (compared to C), ^#^*p* < 0.05 and ^##^*p* < 0.01 (compared to S), ^Δ^*p* < 0.05 and ^ΔΔ^*p* < 0.01 (compared to CIR), and ^†^*p* < 0.05 (compared to SIR).

The differences in TBARS were noticed in nontreated groups. The concentration of TBARS was significantly greater in IR-exposed livers compared to nonischemic group (CIR vs. C, *p* < 0.05). In drug-treated groups, the concentration of TBARS was lower in the ischemic group compared to S (SIR vs. S, *p* < 0.05). Hence, in the STG-treated ischemic group, the level of this parameter was significantly lower compared to the nontreated ischemic group (SIR vs. CIR, *p* < 0.01) (Figure 2(a)).

The activity of PON1 was significantly lower in the nontreated ischemic group than in the nonischemic group (CIR vs. C, *p* < 0.05). Significant difference was also noticed between nontreated and drug-treated nonischemic groups (C vs. S, *p* < 0.05). In IR-exposed groups, the activity of PON1 was statistically higher in STG-treated groups compared to nontreated one (CIR vs. SIR, *p* < 0.05) (Figure 2(b)). There was an inverse relationship between TBARS accumulation and PON1 activity in whole cohort (*ρ* = ‐0.42, *p* = 0.011). The association was stronger in IR animals (CIR and SIR) (*ρ* = ‐0.64, *p* = 0.003).

In IR-exposed groups, the activity of GPx was significantly lower than in nonischemic groups regardless of STG treatment (CIR vs. C, *p* < 0.05; SIR vs. S, *p* < 0.01) (Figure 2(c)). No significant differences in the activity of CAT between experimental groups were noticed. Ischemic-dependent statistically insignificant decrease in the activity of CAT was visible only in nontreated groups (Figure 2(d)).

Ischemic-dependent decrease in the activity of SOD1 in groups nontreated with STG was close to the significance threshold (CIR vs. C, *p* = 0.07). In drug-treated groups, the SOD1 activity was similar regardless of IR. Hence, in the IR conditions in the drug-treated group, the SOD1 activity was significantly higher compared to that in the nontreated group (SIR vs. CIR, *p* < 0.05) (Figure 2(e)).

### 4.3. SOD1 mRNA Expression

In the nonischemic group treated with STG, the expression of SOD1 mRNA significantly increased and was the highest of all examined groups (S vs. C and SIR, *p* < 0.05, and S vs. CIR, *p* < 0.01) (Figure 2(f)).

**Figure 3:**
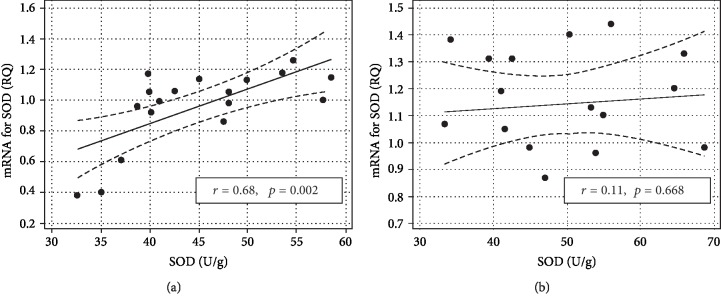
Comparison of correlation between SOD1 expression and activity in control (nontreated) (a) and STG-treated (b) animals. Data presented as regression line with 95% confidence interval (dashed lines).

SOD1 expression positively but moderately correlated with the SOD1 activity in the whole animal cohort (*r* = 0.41, *p* = 0.015). However, this association resulted from a tight relationship between parameters in nontreated animals and was nonexistent in STG-treated ones (Figure 3). Comparison of correlation coefficients between C and CIR groups showed them to be similarly high (*r* = 0.72, *p* = 0.027 and *r* = 0.73, *p* = 0.025, respectively).

### 4.4. Histological Findings

**Figure 4:**
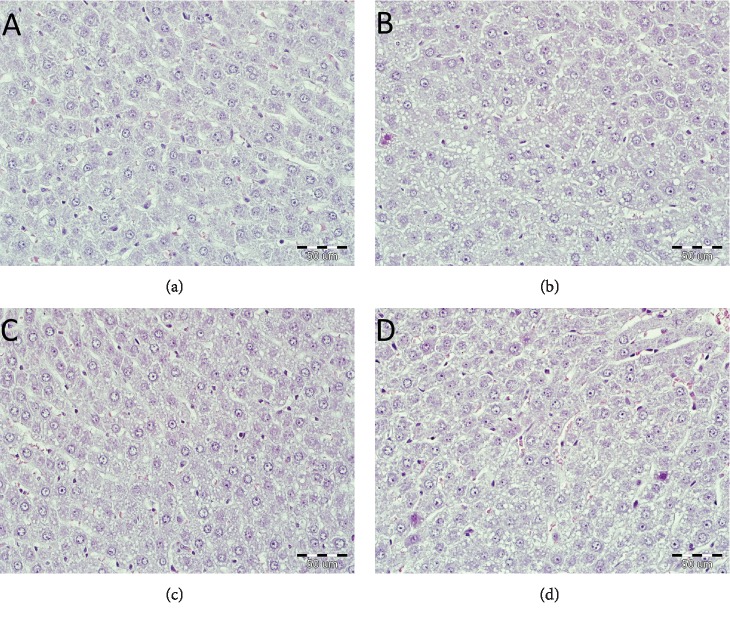
Histopathological examination of liver tissue. Histological examination (stained with hematoxylin-eosin, magnification ×400, bar 50 *μ*m) from group C, rats nontreated and nonsubjected to IR (a); group CIR, rats nontreated and subjected to IR (b); group S, rats treated with STG and not subjected to IR (c); and from group SIR, rats treated with STG and subjected to IR (d). No significant differences in the hepatic structure were seen in both ischemic and nonischemic groups of rats. In drug-treated groups, the percentage of steatosis was statistically higher than that in nontreated groups (CIR vs. SIR, *p* < 0.05, and C vs. S, *p* < 0.01).

No significant differences in the hepatic structure were seen in both ischemic and nonischemic groups of rats. Livers from those groups featured normal architecture, and only slight degree of necrosis and neutrophil infiltration was seen in ischemic groups regardless of the STG treatment. In drug-treated groups, the percentage of steatosis was statistically higher than that in nontreated groups. Described differences were visible in both ischemic and nonischemic groups (CIR vs. SIR, *p* < 0.05, and C vs. S, *p* < 0.01) (Figure 4).

## 5. Discussion

A body of evidence has gathered concerning protective effect of new drugs on hepatic cells during IR, providing rationale for new therapeutic strategies. Among others, glucose-lowering activity of incretins, and hence indirectly of STG, translates into reduced oxidative stress, condition fueled by hyperglycemia [21]. Moreover, STG has been found to be an efficient scavenger of reactive oxygen species (ROS), directly reducing superoxide generation in various organs [22], but the STG effect on oxidative balance in the liver remains unknown. Our work was carried out to understand the effect of STG on the oxidative stress parameters in the liver under IR conditions.

IR resulted in decreased activities of all enzymes examined in our work, but the difference was significant solely in the case of GPx. There is still no consensus regarding IR effect on the SOD1 activity. While some of the authors have shown it to be decreased [23–25], others have found it to be elevated [26] or, as in the case of IR in mice's kidneys, showed the response to be depended on sex [27]. Therefore, to verify SOD1 status, we examined also SOD1 gene expression. Corroborating its reduced activity, relative SOD1 expression was insignificantly decreased by ca 10% upon IR conditions. Interestingly, SOD1 and GPx activities were affected by IR to the same extent (ca 18%), which corresponds well with 20% drop in glutathione level, an electron donor in the reactions catalyzed by GPx, reported recently by Weng et al. [23].

STG has been shown to increase the SOD, CAT, and GPx activities in kidneys [16], organs with the highest DPP-4 activity [21]. However, this effect was observed only in rats with diabetes. In the kidneys of healthy animals, the SOD activity decreased slightly, the CAT activity increased slightly, and the GPx activity remained unchanged [28]. These observations may imply that beneficial effects of drug are displayed solely in the presence of an additional factor increasing the oxidative stress. IR is another condition, in which STG treatment has been associated with improved antioxidant status both at systemic level [29] and locally in myocardium [24], kidney [28], and hippocampus [30]. In our work, carried out on rat livers, the protective effect of STG was visible only under IR conditions. In the ischemic liver, the CAT and SOD1 activities decreased insignificantly in untreated groups but remained unchanged in the STG group. Therefore, in the case of SOD1, significant differences were observed between treated and untreated groups.

Interestingly, SOD1 expression in our STG-treated nonischemic livers was significantly upregulated, which, however, did not translate into increased enzyme activity. The discrepancy between drug effect on enzyme gene expression and activity could also be found in the case of GPx in kidneys: its mRNA expression decreased [31] but enzymatic activity increased [16]. One must keep in mind that many factors may affect translation and thus up- or downregulate expression on protein level as compared to mRNA copy numbers or, in the case of the enzymes, also modulate their activity. Indeed, antioxidant enzymes are sensitive to oxidative damage themselves. Moderate oxidative imbalance stimulates expression of antioxidant enzymes through activation of *Nrf2*, but it may also diminish their activity by causing oxidative modifications, e.g., oxidation of cysteine thiols. However, the correlation analysis of SOD1 expression and activity showed them to be tightly and positively correlated with nontreated animals, but it was completely abolished in STG-treated animals. Taken together, results showed that STG differently affected SOD1 at transcriptional and enzyme activity levels and was responsible for the disruption of the association between both parameters.

*Nrf2* is a key transcription factor responsible for induction of antioxidant enzymes synthesis. It was recently demonstrated that STG downregulated the expression of Nrf2 in rat kidneys, with concomitant upregulation of its inhibitor—Keep1. These findings imply that, at least in kidneys, antioxidant action of STG is associated rather with direct reduction of intracellular ROS or with increased stability of GLP-1. Since we observed upregulation of SOD1 expression in the liver, it would be of interest to investigate the drug effect on hepatic *Nrf2* [31].

A reduction of hepatic PON1 activity during lipid peroxidation and liver damage was an early occurring phenomenon, what was shown in animal models [32]. Accordingly, a drop in the PON1 activity has accompanied also hepatic IR injury [33, 34]. Corroborating these observations, we found PON1 to be significantly decreased in the ischemic nontreated group. In line with the beneficial effect on the oxidative balance attributed to STG during IR [25, 28, 30], the enzyme activity in the ischemic drug-treated group was restored and significant differences were observed between treated and untreated groups. However, the PON1 activity was diminished in drug-treated animals not subjected to IR. These results seem to imply that STG *per se* might have a negative impact on the enzyme. A positive correlation between the PON1 activity and insulin resistance was shown [35, 36]. The high glucose concentration was shown to induce activation of specific protein (SP)-1 [37, 38], a transcription factor activating the PON1 promoter in cultured hepatocytes [37]. STG increasing the sensitivity of tissues to insulin may negatively affect the expression of PON1 in the liver. However, functional studies are needed to determine the exact effect of STG on the enzyme.

Simultaneously with the decreased activity of PON1 in untreated ischemic livers, a significant increase in TBARS concentration was observed. Our findings corroborate earlier reports on increased accumulation of lipid peroxidation products in response to IR injury in the liver [9, 26, 39–42]. STG has been reported to possess antioxidant properties manifested, among others, by its capability to limit lipid peroxidation [22]. Under IR conditions, the effect of STG lowering MDA accumulation has previously been observed in the brain [30], kidney [28], and heart [25]. We showed that MDA formation was reduced after STG treatment also in livers subjected to IR. However, without pathology such as IR or diabetes, STG seems to have an opposite effect and increase the accumulation of TBARS, what was also noticed by other authors [29, 43].

While unexpected, this finding is consistent with the diminishing effect of the drug on PON1 and a close negative correlation between liver PON1 activity and accumulation of TBARS observed in our study as well. Therefore, considering the role of PON1 in protection against lipid peroxidation [7, 8], enhanced lipid peroxidation in STG-treated animals might directly result from drug-induced inhibition of PON1. Surprisingly, in studies evaluating beneficial effects of various drugs on IR injury, a control drug-treated group has frequently been missing, also in the case of STG [25, 30].

The histological evaluation of liver specimens showed no significant differences in IR-dependent liver structure. Only slight degree of necrosis and neutrophil infiltration was observed in ischemic groups. To monitor the liver dysfunction, which intensifies with the duration of IR, we determine the activity of aminotransferases—enzymes released from damaged hepatocytes [44, 45]. As in our previous works [46–48], we have also demonstrated in this work that the aminotransferase activity was significantly increased, most pronounced in the nontreated ischemic group. Therefore, despite a minimal histological abnormality, we may suspect a low-degree liver injury evoked by IR.

The STG is minimally metabolized in the liver, and over 80% is excreted with the urine in unaltered form [49]. Therefore, this medicine has a safe and beneficial pharmacokinetics even in patients with liver damage. The STG safety profile assessed in many studies is rather good, and the risk of liver damage does not increase. In clinical trials, the use of STG alone or in combination with other oral antidiabetic agents did not cause alterations in AST or ALT [50, 51]. There are only a few cases of serious liver damage after STG treatment [52, 53]. In our work, the effect of STG therapy on liver damage probably depends on the initial conditions. The higher steatosis rates observed in STG-treated groups suggested detrimental effects of the drug itself during chronic treatment. On the other hand, the aminotransferase activity before and during IR in the drug-treated group was lower at all time points, and in the 24-hour reperfusion, the difference was significant. Such results, however, indicate a protective effect of STG, which is more visible in harmful conditions, such as IR in our case.

## 6. Conclusions

Summing up, the action of STG strongly depends on additional factors increasing the oxidative stress, such as IR. In the nonischemic group, the effect of STG on oxidative parameters was invisible or even negative. However, under IR, the action of this drug is beneficial. Also, despite the small degree of steatosis, the aminotransferase activity analysis does not suggest any hepatotoxic action of STG. Contrarily, even a slight protective effect of this drug was seen, especially in IR conditions.

Although significant results have been obtained in this work, the limitations of this study should be considered. In our work, we observed the effect of STG only on the parameters of oxidative stress in the rat liver subjected to IR. It will be important in further studies to also examine other markers of liver injury during IR, especially the ones associated with inflammation and apoptosis. To better explain the STG's mechanism of protective action, we also plan to explore a grip point of this drug. Furthermore, we are now aware, that by extending the duration of administration and using higher STG doses, a more pronounced effect of this drug may be achieved. And finally, the limitations of a partial liver ischemia model used in this work need to be considered. During the induced ischemia of the middle and left lateral lobe, there is an increased blood flow in the rest of the liver, which can affect liver function and regeneration. Exchange of oxygen from perfused to ischemic part of the liver may affect the values of oxidative stress parameters obtained in this experiment.

## Data Availability

The laboratory data used to support the findings of this study are available from the corresponding author upon request.

## Abbreviations

ALT: Aminotransferase alanine
AST: Aminotransferase aspartate
CAT: Catalase
DPP-4: Dipeptidyl peptidase-4
GIP: Glucose-dependent insulinotropic polypeptide
GLP-1: Glucagon-like peptide-1
GPx: Glutathione peroxidase
H2O2: Hydrogen peroxide
IR: Ischemia/reperfusion
MDA: Malondialdehyde
PON1: Paraoxonase-1
ROS: Reactive oxygen species
SOD: Superoxide dismutase
STG: Sitagliptin
TBARS: Thiobarbituric acid reactive substances.
